# Supplementary material for: From sequence to enzyme mechanism using multi-label machine learning
Source: BMC Bioinformatics. 2014 May 19;15:150. doi: 10.1186/1471-2105-15-150 (PMC4229970; doi:10.1186/1471-2105-15-150)
Supplement: Additional file 2 — Java code of ml2db. Additional file ml2db_code.tar.gz contains the Java source code to run the multi-label machine learning experiments and save the results to database. The code’s Javadoc is included. [file 1471-2105-15-150-S2.zip › additional file 2/ml2db/ml2db/doc/main/ml2db/mulan/classifier/lazy/BRkNNVisible.html]

BRkNNVisible


JavaScript is disabled on your browser.


- Overview
- Package
- Class
- Use
- Tree
- Deprecated
- Index
- Help

- Prev Class
- Next Class

- Frames
- No Frames

- All Classes

- Summary:
- Nested |
- Field |
- Constr |
- Method

- Detail:
- Field |
- Constr |
- Method


main.ml2db.mulan.classifier.lazy

## Class BRkNNVisible

- java.lang.Object
- - mulan.classifier.MultiLabelLearnerBase
  - - mulan.classifier.lazy.MultiLabelKNN
    - - main.ml2db.mulan.classifier.lazy.BRkNNVisible

- All Implemented Interfaces:
  :   java.io.Serializable, mulan.classifier.MultiLabelLearner, weka.core.TechnicalInformationHandler

  Direct Known Subclasses:
  :   BRkNNFewInstances

  ---

    

  ```
  public class BRkNNVisible
  extends mulan.classifier.lazy.MultiLabelKNN
  ```

  A version of BRkNN Mulan 1.4 that exposes more methods as public, and has
  more logging and debugging capabilities.

  Version:
  :   16 Oct 2012

  Author:
  :   Luna De Ferrari ldf(at)st-andrews.ac.uk or
      luna.deferrari@st-andrews.ac.uk

  See Also:
  :   Serialized Form

- - ### Nested Class Summary

    Nested Classes

    | Modifier and Type | Class and Description |
    | --- | --- |
    | `static class` | `BRkNNVisible.ExtensionType` The two types of extensions |
  - ### Field Summary

    - ### Fields inherited from class mulan.classifier.lazy.MultiLabelKNN

      `WEIGHT_INVERSE, WEIGHT_NONE, WEIGHT_SIMILARITY`
  - ### Constructor Summary

    Constructors

    Constructor and Description

    `BRkNNVisible()`

    Default constructor

    `BRkNNVisible(int numOfNeighbors)`

    A constructor that sets the number of neighbors

    `BRkNNVisible(int numOfNeighbors,
    BRkNNVisible.ExtensionType ext)`

    Constructor giving the option to select an extension of the base version
  - ### Method Summary

    Methods

    | Modifier and Type | Method and Description |
    | --- | --- |
    | `java.io.File` | `getFile()` |
    | `weka.core.TechnicalInformation` | `getTechnicalInformation()` |
    | `java.lang.String` | `globalInfo()` |
    | `void` | `printNeighbours(weka.core.Instance instance, weka.core.Instances neighbours)` |
    | `void` | `setCvMaxK(int cvMaxK)` set the maximum number of neighbors to be evaluated via cross-validation |
    | `void` | `setkSelectionViaCV(boolean flag)` |

    - ### Methods inherited from class mulan.classifier.lazy.MultiLabelKNN

      `isUpdatable, setDfunc, setDistanceWeighting`
    - ### Methods inherited from class mulan.classifier.MultiLabelLearnerBase

      `build, getDebug, makeCopy, makePrediction, setDebug`
    - ### Methods inherited from class java.lang.Object

      `equals, getClass, hashCode, notify, notifyAll, toString, wait, wait, wait`

- - ### Constructor Detail


    - #### BRkNNVisible

      ```
      public BRkNNVisible()
      ```

      Default constructor


    - #### BRkNNVisible

      ```
      public BRkNNVisible(int numOfNeighbors)
      ```

      A constructor that sets the number of neighbors

      Parameters:
      :   `numOfNeighbors` -


    - #### BRkNNVisible

      ```
      public BRkNNVisible(int numOfNeighbors,
                  BRkNNVisible.ExtensionType ext)
      ```

      Constructor giving the option to select an extension of the base version

      Parameters:
      :   `numOfNeighbors` -
      :   `ext` - the extension to use (see `BRkNNVisible.ExtensionType`)
  - ### Method Detail


    - #### getFile

      ```
      public java.io.File getFile()
      ```

      Returns:
      :   m\_file


    - #### getTechnicalInformation

      ```
      public weka.core.TechnicalInformation getTechnicalInformation()
      ```

      **Specified by:**
      :   `getTechnicalInformation` in interface `weka.core.TechnicalInformationHandler`

      **Specified by:**
      :   `getTechnicalInformation` in class `mulan.classifier.MultiLabelLearnerBase`


    - #### globalInfo

      ```
      public java.lang.String globalInfo()
      ```

      **Specified by:**
      :   `globalInfo` in class `mulan.classifier.MultiLabelLearnerBase`


    - #### printNeighbours

      ```
      public void printNeighbours(weka.core.Instance instance,
                         weka.core.Instances neighbours)
      ```


    - #### setCvMaxK

      ```
      public void setCvMaxK(int cvMaxK)
      ```

      set the maximum number of neighbors to be evaluated via cross-validation

      Parameters:
      :   `cvMaxK` -


    - #### setkSelectionViaCV

      ```
      public void setkSelectionViaCV(boolean flag)
      ```

      Parameters:
      :   `flag` - if true the k is selected via cross-validation


- Overview
- Package
- Class
- Use
- Tree
- Deprecated
- Index
- Help

- Prev Class
- Next Class

- Frames
- No Frames

- All Classes

- Summary:
- Nested |
- Field |
- Constr |
- Method

- Detail:
- Field |
- Constr |
- Method
